# Application of Amberlite IRA 402 Resin Adsorption and Laccase Treatment for Acid Blue 113 Removal from Aqueous Media

**DOI:** 10.3390/polym13223991

**Published:** 2021-11-18

**Authors:** Nicoleta Mirela Marin, Ioana Stanculescu

**Affiliations:** 1 National Research and Development Institute for Industrial Ecology ECOIND, Street Podu Dambovitei No. 57-73, District 6, 060652 Bucharest, Romania; 2 Department of Physical Chemistry, Faculty of Chemistry, University of Bucharest, 4-12 Regina Elisabeta Bd., 030018 Bucharest, Romania; 3 Horia Hulubei National Institute for Physics and Nuclear Engineering, IRASM, 30 Aleea Reactorului, 077125 Magurele, Romania; 4 Faculty of Applied Sciences, Reactor Institute Delft, TU Delft, Mekelweg 15, 2629 JB Delft, The Netherlands

**Keywords:** diazo dye, ion exchange mechanism, polymeric functionalized resin, pilot laboratory experiment, laccase

## Abstract

Despite Acid Blue 113 (AB 113)’s extensive use and negative environmental impact, very few studies have focused on its efficient and environmentally friendly removal. This research aims the removal of AB 113 from environmental aqueous media and its consequent enzymatic biodegradation. A strongly basic anion exchange resin in Cl^−^ form, Amberlite IRA 402 (IRA 402(Cl^−^)) was used for AB 113 adsorption and a laccase was used to further biodegrade it. For the first time, two novel, efficient and environmentally friendly physical–chemical and biological assays for AB 113 wastewater removal and subsequent biodegradation were combined. The adsorption of AB 113 onto IRA 402(Cl^−^) was tested in batch and continuous flux modes. Influence of contact time, concentration and desorption in acidic media were evaluated. The kinetic data were best modulated by the Lagergren model with R^2^ = 0.9275. The Langmuir isotherm model best fitted the experimental data, and the maximum adsorption capacity was 130 mg/g. Dye, resin and AB113 loaded resin were characterized by thermogravimetry and FTIR to evaluate their physical chemical properties modification. Based on the performed studies, a consecutive methodology is proposed, incorporating the ion exchange process in the first stage and the biodegradation process in the second. Thus, in the second stage the residual concentration of AB 113 is reduced by an efficient bio-degradation process produced by the laccase at pH = 4.

## 1. Introduction

Synthetic dyes are necessary in various important fields, such as the textile, leather, paper and varnish and paint industries. It is estimated that approximately 700,000 tons of dyes are manufactured each year [1]. Among the above-mentioned industries, the textile industry releases the highest concentration of dye in its effluent. Approximatively 54% of the dyes released in the environment around the world come from the textile industry, 21% from varnishes and paints, 10% from paper and cellulose, 8% tanning industry and 7% from paints and dye-producing. Moreover, high concentrations of dyes in effluents from various activities associated with these industries are undesired [2,3]. For various dyeing processes in the textile industry, specific mixtures of dyes and chemicals dissolved in water are required. Once the coloring process is completed, the remaining wastewater must be treated before being disposed into the environment without causing pollution. Dyes are discharged into effluent because they are weakly retained by fabric thread fibers by chemical bonds or by weak physical interactions. The color is the most obvious indicator of water dye pollution. Approximatively, 80% of the dye molecules are adsorbed by the textile fibers, with 20% remaining in solution [4,5]. The effluents obtained after the dyeing process should be treated before discharge to protect ecosystems and the lives of animals and people. Dye molecules have the role of giving color to the material on which they are applied. This is possible because dyes may bind to any accessible material surface, through physical interactions or chemical bonds [6,7]. Initially, natural dyes were obtained in small quantities from insects, plants, fruits and vegetables, but they have the disadvantage of fading after wearing, repeated washing and sunlight exposure. Due to growing demand in various industries, synthetic dyes have been used in high quantities over the years. In the year 1856, a wide range of synthetic dyes, including the azo dye class, was invented by WH Perkins. Thus, this discovery solved the problem of natural colors, but new issues have emerged, e.g., industries using synthetic dyes must reduce their concentration in wastewater before disposal in surface waters [8]. Synthetic dyes may exist in solution in the dissociated form, when their structures have ionizable groups (either acidic or basic). In view of all the above, wastewater containing synthetic dyes may not be discharged into the environment without treatment [9,10,11,12,13,14]. Given their major toxicity, an ideal method should be able to remove dyes in high concentrations from wastewater in a very short time without producing secondary pollution. Among the multitude of conventional methods, the ion exchange process offers one of the best removal efficiencies for azo dyes [15,16]. The first synthetic ion exchange resins of phenol-formaldehyde type, modified with sulfonic groups were obtained as early as 1935 by Adams and Holmes. Subsequent research focused on the use of ion exchange hydrocarbon structure resins modified by styrene divinylbenzene copolymerization. Sulfonic or carboxyl groups attached by synthetic methods yield cation exchangers and amine groups attached generate anion exchangers. These ion exchangers represent 90% of resins used in analytical studies [17]. In general, the synthesis of a modern ion exchanger is performed in two steps. In the first step, a spherical granule is obtained by copolymerizing styrene with divinylbenzene in water, which and is currently the most widely used industrial process for the manufacture of polystyrene resins. This copolymerization step is also used in the synthesis of non-functionalized polymers used in ion exchange equilibria. In the second stage of the synthesis, the ionic functional groups are grafted. They contain a fixed group that is not exchangeable and a counterion that participates in the ion exchange equilibrium. The functional groups may be acidic or basic and may be grafted onto the polymer substrate by synthetic methods or may pre-exist in the structure of one of the monomers. Anion exchange resins have positively charged ionizable functional groups and exchange negatively charged counterions in solution [18]. The use of ion exchange resins in wastewater treatment has many advantages. They allow the elimination of almost all pollutants found as cationic or anionic species, whether organic or inorganic. The popularity of ion exchange resins has remained constant over time, as this separation method can be applied in environmental depollution methodologies (water, air, soil and waste), biotechnology, agriculture and pharmacy, as well as in the dye industry. Another important advantage is related to the wide variety of resins available in the market, some of them being specific for certain applications [19]. Ion exchange resins are the alternative for ionizable pollutants removal from water due to their good exchange capacity and selectivity. Thus, IRA 402 was chosen owing its specificity for high molecular weight compounds. The aims of this study were to remove the AB 113 onto IRA 402 resin and subsequently, to apply a biodegradation process for improving water treatment processes. To achieve these aims, the following steps were tested: (i) evaluation of contact time between AB 113 and IRA 402; (ii) the influence of AB 113 concentrations; (iii) the desorption mechanism of AB 113 from IRA 402; (iv) adsorption kinetics and isotherm studies; (v) development a laboratory pilot experiment for continuous-flow AB 113 removal; (vi) evaluation of AB 113 dye biodegradation with Laccase and (vii) evaluation of the thermal stability of AB 113 by TG and of dye–resin physical–chemical interactions by FTIR studies with a view to unravelling the retention mechanism.

## 2. Materials and Methods

### 2.1. Chemicals

IRA 402(Cl^−^), a strongly basic anion exchanger in Cl^−^ form, yellowish, moist, with 1.4 mol/L ion exchange capacity, in spheres of 0.3–1.18 mm was obtained from Merck, Darmstadt, Germany. AB 113 powder was obtained from Sigma Aldrich, Shanghai, China. The stock and working solutions of AB 113 were prepared in ultrapure water obtained with an Ultra-Clear system, Richfield, UT, USA. The 37% HCl, was supplied by Merck, Darmstadt, Germany. Laccase from *Trametes versicolor* (0.5 U/mg, light brown, powder) was from Sigma Aldrich, Steinheim, Germany.

### 2.2. Equipment 

The synthetic solutions of AB 113 used for adsorption, desorption and for biodegradation experiments were analyzed using a UV-Vis spectrometer from Hach Lange, Germany) in 1 cm quartz cells using the 200–800 nm. The spectra were recorded at 100 nm/min scanning speed for the ultrapure water in the adsorption experiment, acidic solutions for desorption experiment and dye-tampon buffer for biodegradation experiment. The pH buffer was measured with HI 255 Combined Meter (pH/mV & EC/TDS/NaCl) from Hanna Instruments, Nijverheidslaan, Belgium. The mass of IRA 402 and AB 113 powder was weighted with a XT220A analytical balance from Precisa Gravimetrics, Dietikon, Switzerland, with a precision of ±0.0004 g. For thermal analysis a STA 409 PC Luxx simultaneous thermogravimeter-differential scanning calorimeter TG/DSC from Netzsch, Selb, Germany was employed to verify AB 113 dye stability. FTIR spectra were acquired with a Vertex 70 spectrometer from Bruker, Ettlingen, Germany in transmittance mode in the 4000–400 cm^−1^ range with 4 cm^−1^ resolution and 32 scans. 

### 2.3. Activation of IRA 402 Resin

The IRA 402(Cl^−^) resin may contain impurities, so it is necessary to remove them and activate it. First, 100 g of IRA 402(Cl^−^) was washed with ultrapure water. Second, the swollen resin was transferred in a 1000 mL Berzelius flask and stirred for 2 h at 100 rpm with horizontal stirrer mixer at laboratory temperature with 500 mL 1 M HCl. The resin activated in Cl^−^ form was washed with ultrapure water until the pH of supernatant was neutral and dried for a few days in a desiccator up to the constant mass of IRA 402(Cl^−^) detection [15,16,20]. 

### 2.4. Batch Kinetic and Equilibrium Procedures

The influence of the contact time on the adsorption of the AB 113 dye onto IRA 402(Cl^−^) was studied by varying the contact between liquid and solid phases from 0 to 135 min, using solutions of concentrations of 2365 mg/L AB 113 and 0.5 g of IRA 402(Cl^−^).

Equilibrium ion exchange experiments regarding the AB 113 dye adsorption onto strongly basic anionic resin Amberlite IRA 402 were performed using the horizontal stirring method, by adding 0.5 g of strongly basic anionic resin Amberlite IRA 402(Cl^−^), in conical glasses over which various AB 113 dye solutions were added. The conical glasses were fixed in the mechanical stirrer horizontally and stirred at a constant speed of 175 rpm at T = 25 ± 2 °C for 90 min. After stirring, the samples were filtered into 50 mL volumetric flasks, and the concentration at equilibrium of AB 113 in the filtered solution was determined by UV-VIS molecular absorption spectrometry.

### 2.5. Desorption Experiments

For the desorption experiments, approximately 0.5 g of IRA 402 resin loaded with AB 113 at equilibrium (110 mg AB 113/g of IRA 402) for each HCl concentration tested was weighed into a 100 mL conical flask. Desorption of the resin loaded was evaluated in acid solutions. 20 mL of 1, 2, 3, 4, 5, 6 and 7 M HCl were added over the 0.5 g resin samples loaded with AB 113. The mixtures obtained were stirred for 90 min at 175 rpm. After 30 min stirring the mixture was filtered and the solutions were collected and analyzed spectrometrically, measuring the AB 113 concentration. 

### 2.6. Experiments of Pilot Laboratory Test

The pilot installation consisted of a supply vessel, peristaltic dosing pump and a glass column with height and inner diameter of 25 × 2.5 cm. The column was packed with 32 g of IRA 402(Cl^−^) before being swollen in water for 24 h. Afterwards, the swollen resin was quantitatively transferred inside the column obtaining a 10 cm height of the bed material in the column. The experiment performed in the laboratory was designed in the down flow mode of the influent (aqueous dye solution of 56 mg/L) through the resin bed, for 600 h with a flow rate (Q) of 0.7 mL/min fixed with the peristaltic pump. The number of rotations was established and kept constant at 10 rpm for all the pilot laboratory experiments. A polypropylene sieve was also mounted at the bottom of the column as a support for resin bed to keep the resin bed stabile. The volume of treated aqueous solution was measured using a graded cylinder.

### 2.7. Experiments for Enzymatic Degradation

The enzymatic biodegradation reaction was evaluated at pH = 4 using citric acid/sodium hydroxide/hydrogen chloride buffer solution obtained from CertiPUR. The biodegradation tests were performed directly in the spectrometer quartz cuvettes at room temperature, in dark and static conditions. The biodegradation reaction consisted of mixing 1000 µL buffer pH = 4, 1000 µL AB 113 dye solution of 0.00007 M and 500 µL laccase solution of 0.5 U/mL.

### 2.8. Statistical Analysis

All experiments were performed in triplicate and average values are presented. The fit of the experimental data was based on these values. The linear dependence between the spectrum area (A) and the dye concentration (C) was evaluated in the concentration range 7.6–61.1 mg/L. The linear regression equation was obtained as A = 3.6585 C + 12.207 which was used to determine the dye concentration in the supernatant after contact with the resin. The limit of detection of the spectrometric method was 1.48 mg/L and the limit of determination was 4.92 mg/L. The precision of the spectrometric method, evaluated by the relative standard deviation (RSD%), was 0.58%, and the accuracy of the method, evaluated by the mean recovery yield, was 98.9% with an RSD (%) of 1.094%.

## 3. Results and Discussions

### 3.1. Ion Exchange Equilibrium of AB 113 Dye onto IRA 402(Cl^−^)

In aqueous solution the sulfonic groups of the AB 113 exist in dissociate form. The ion exchange process takes place due to the interactions that occur between the dissociated sulfonic groups of AB 113 and the quaternary ammonium groups of the anion exchanger in the Cl^−^ form one. Therefore, the ion exchange process is the main mechanism that governs adsorption process, but surface interactions mediated by van der Waals and π–π interactions, which occur between aromatic structure of AB 113 and IRA 402(Cl^−^), must also be taken into account. Similar results have been presented in the literature [15,16,21,22].

### 3.2. Batch Kinetic and Equilibrium Studies

**Figure 1:**
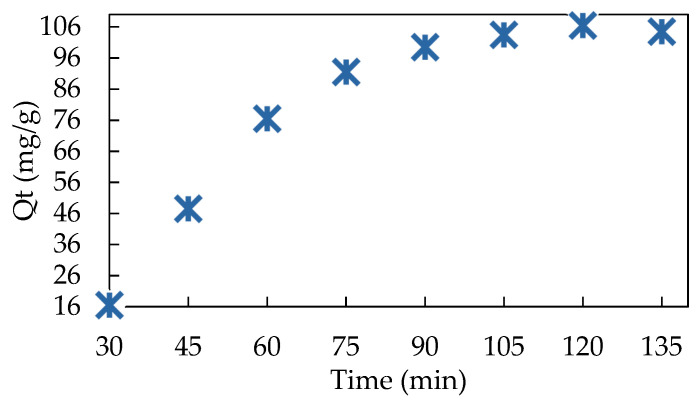
Experimental data of contact time between AB 113 onto IRA 402 mass. The values are the average of three replicates with RSD% below 8% for all experiments.

The results obtained reveals that the adsorption process takes place in two stages. In the first step, from 0–90 min, the adsorption process was fast. Subsequently, in the second step, the adsorption rate decreased and the amount (Q_t_) retained varied insignificantly from 103.5 up to 104.5 mg/g (Figure 1). Similar studies regarding the influence of contact time on Congo Red dye retention on Amberlite IRA 400 resin have been performed [23]. It was observed that the adsorption of Congo Red at 50 mg/L was achieved quickly, compared with 70 and 100 mg/L concentrations. At the same time, 175 min was necessary to attain equilibrium for complete dye removal. The retention of Acid Orange 10 dye by Amberlite IRA 400 resin was studied in our previous work, its adsorption being achieved in 35 min [15]. In another previous study, Acid Blue 193 was removed in 60 min from Amberlite IRA 400 [16].

The behavior of the reaction rate can be analyzed using kinetic adsorption models. The kinetic trend was determined by fitting the adsorption capacity (Q_t_) vs. the adsorption time (t) in Figure 1. From this investigation, Q_t_ was calculated with the following equation (eq):

(1) $$Q_{t}=\frac{\left(C_{i}{-C}_{t}\right)V}{m}$$

where: C_i_ and C_t_ (mg/L) represent the AB 113 concentration at the beginning of the experiment and at 30, 45, 60, 75, 90 and 105 min; m (g) is the mass of IRA 402(Cl^−^), V (L) is the volume of AB 113 solution used in the experimental study. First order kinetic model (PFO), namely, Lagergren, 1898, is described by the equation

(2) $$\log\left(Q_{e}{-Q}_{t}\right){=\mathrm{logQ}}_{e}-\left(\frac{K}{2.303}\right)t$$

and the second-order kinetic model, proposed by Ho and McKay, 1998, was modulated using the following linear Equations (3a)–(3d):

(3a) $$\frac{t}{Q_{t}}=\frac{1}{K_{2}Q_{e}2}+\frac{t}{Q_{e}};\mathrm{Type}1$$

(3b) $$\frac{1}{Q_{t}}=\frac{1}{Q_{e}}+\frac{1}{K_{2}Q_{e}2}\frac{1}{t};\mathrm{Type}2$$

(3c) $$Q_{t}=Q_{e}-\frac{1}{K_{2}Q_{e}}\left(\frac{Q_{t}}{t}\right);\mathrm{Type}3$$

(3d) $$\frac{\mathrm{Qt}}{t}=K_{2}(Q_{e}\times Q_{e})-K_{2}Q_{e}\;Q_{t};\mathrm{Type}4$$

**Figure 2:**
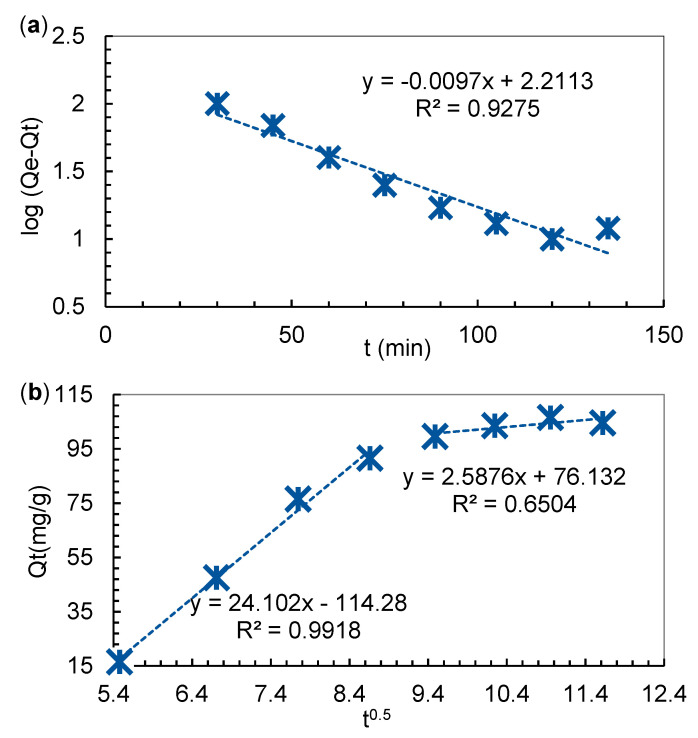
Experimental kinetic data fit of: (**a**) PFO and (**b**) Weber–Morris.

The kinetic constants including Q_eq_, Q_t_, K_1_, K_2_ and R^2^ respectively for the AB 113 adsorption onto IRA 402(Cl^−^) mass were determined and are presented in Table 1. Applicability of the kinetics model was carried out by correlation coefficient (R^2^) evaluation of obtained linear regression. Moreover, analyzing experimental data presented in Table 1, obtained when fitting kinetics results, it was found in Figure 2a that R^2^ for the PFO model are greater than for PSO models modulated as: t/Q_t_ vs. t (Figure S1); 1/Q_t_ vs. 1/t (Figure S1); Q_t_ vs. Q_t_/t (Figure S1) and Q_t_/t vs. Q_t_ (Figure S1). The determined values of Q_eq_ are 162.7 mg/g for PFO and 626; 97.1; 27.9 and 0.07 mg/g for PSO model when Equation (3a) up to Equation (3d) were applied. The Q_t_ results of type 2 PSO model are close with the Q_t_ experimental value determined at influence contact time (see Figure 1 and Table 2).

The intra-particle diffusion model proposed by Weber and Morris may be expressed with the following Equation:

(4) $$Q_{t}=K_{\mathrm{id}}{\left(t\right)}^{0.5}+C$$

where C is the quantity of AB 113 adsorbed at the surface of the IRA 402 resin mass and K_id_ (mg/g min^0.5^) represents the intraparticle diffusion rate constant. From the slope and intercept of Qt vs. t^0.5^, the values of K_id_ and C were obtained by applying the Weber–Morris Equation (4). Their values are presented in the linearized graph of Figure 2b and in Table 1.

One may observe from Figure 2b that the linear graph is divided into two stages, in the first stage, AB 113 was retained at the surface of the particle only in film diffusion, and the second stage represents the adsorption equilibrium, so the Weber–Morris model would not be applied. Thus, as can be seen in Figure 2b, the higher value of slope determined for the first stage of adsorption compared to the slope determined for the second stage suggests that the ion exchange resin ionizable groups are found on the surface of the resin particle and are much more accessible. Therefore, there is a limited diffusion of AB 113 dye molecules in the porous structure that does not influence the adsorption process [24].

### 3.3. Batch Adsorption Studies

**Figure 3:**
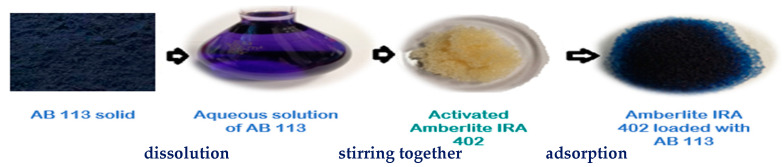
Image of batch adsorption process of AB 113 onto Amberlite IRA 402.

AB 113 and IRA 402(Cl^−^) ion exchange process steps are designed in Figure 3.

**Figure 4:**
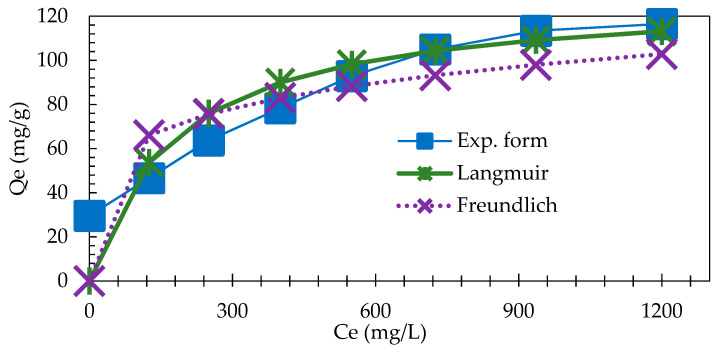
Experimental isotherm regarding AB 113 adsorption onto IRA 402(Cl^−^) mass; the values presented are the averages of three replicates with RSD% below 8% for all experiments.

The quantities of AB 113 retained in IRA 402(Cl^−^) mass at equilibrium were calculated using following equation:

(5) $$Q_{\mathrm{eq}}=\frac{\left(C_{i}-C_{\mathrm{eq}}\right)V}{m}$$

as 29.6, 46.7, 63.7, 78.3, 92.8, 105 and 117 mg/g for initial concentrations of AB 113 of 296, 592, 887, 1183, 1479, 1775 and 2070 mg/L, respectively (Figure 4). These results suggest that the AB 113 adsorption decreased when the saturation of IRA 402(Cl^−^) resin was reached. 

In another study, AB 113 was removed from aqueous solutions using nonconventional adsorbents such as: activated red mud, where 83 mg/g AB 113 was removed [25]; leaves of almond treated with NaOH and modified with surfactant removed 25.5 and 97.09 mg/g of AB 113, respectively [26]; overripe *Cucumis* peel removed 97.09 mg/g of AB 113 [27] and mesoporous activated carbon and rubber tire removed 7.84 and 113 9.72 mg/g of AB 113, respectively [28]. Similar research on adsorption capacity of different type of strongly basic ion exchange resins are presented in the Table 2.

**Table 2:** Polymeric resin used to remove dyes from water.

Resin	Azo Dye	Q_eq_ (mg/g)	References
Strongly basic anion exchange resin Amberlite IRA-958	Acid Orange 7	1370	[29]
Amberlite IRA-900 (Strongly basic, type 1—N^+^(CH_3_)_3_)	Tartrazine	57.6	[30]
Amberlite IRA-910 Strongly basic, type 2—N^+^(CH_3_)_2_C_2_H_4_OH		44.6	
Polystyrene anion-exchange resin SAER	Acid Orange 10	670	[31]
Polyacrylic anion-exchange resin AAER		855	
Amberlite IRA67	Acid Orange 7	168	[32]
Amberlite IRA-458		1211	
Purolite A-520E	Acid Blue 29	321,5	[33]
Amberlite IRA 900	Reactive Black 5	1352	[34]
Amberlite IRA 402(Cl^−^)	AB 113	117	This study

#### 3.3.1. Adsorption Isotherm Study

##### Langmuir Isotherm 

In general, adsorption study is modeled with isotherms described by mathematical equations. Isotherm studies are useful to detect the concentrations ratio of AB 113 between solid and liquid phases and to provide important information about those, when equilibrium is reached [35]. Thus, the adsorption process of AB 113 onto IRA 402(Cl^−^) resin, applying the Langmuir and Freundlich isotherm model, was evaluated. The Langmuir isotherm describes the quantitative adsorption of AB 113 on homogeneous surface of the IRA 402(Cl^−^) resin and was modulated using the following Equation:

(6) $$\frac{C_{\mathrm{eq}}}{\mathrm{Qe}}=\frac{1}{{\mathrm{bQ}}_{0}}+\frac{C_{\mathrm{eq}}}{Q_{0}}\;\;$$

**Figure 5:**
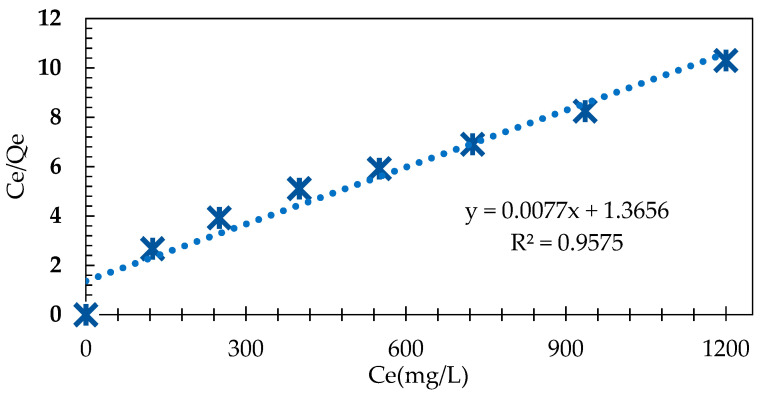
Linear regression of the Langmuir isotherm regarding AB 113 adsorption onto IRA 402(Cl^−^) mass.

From the slope and intercept of the linear plot $\frac{C_{\mathrm{eq}}}{\mathrm{Qe}}$ vs. C_eq_, the values of $\frac{1}{Q_{0}}$ and $\frac{1}{{\mathrm{bQ}}_{0}}$ were determined (see Table 3). As one may observe, the experimental data obtained according to the Langmuir isotherm fits very well our results (Figure 5). The IRA 402(Cl^−^) resin affinity was estimated based on the constant *b* that represents the adsorption energy involved in the adsorption process and Q_m_ is maximum monolayer adsorption capacity of IRA 402(Cl^−^) resin. The value of R^2^ obtained from the graphical representation of the $\frac{\mathrm{Ce}}{\mathrm{Qe}}$ vs. C_eq_ (mg/L) was 0.9575 and the Q_m_ obtained was 130 mg/g. The R_L_ constant value of the present investigation is 0.60 which, being less than 1, suggests favorable AB 113 adsorption onto IRA 402(Cl^−^). The high value of R^2^ showed that the adsorption of AB 113 is very well described by the Langmuir isotherm. Similar results have been reported in literature when the Langmuir isotherm has been applied to fitting experimental data. Polska et al. showed that the adsorption data of Acid Red 18, Reactive Blue 21 and Direct Yellow 142 onto Amberlite IRA 478 obeyed the Langmuir isotherm [21]. In addition, Saruchi et al. used a synthesized hybrid ion exchanger for rhodamine B adsorption. It was observed that the Langmuir model fitted experimental data very well and the maximum adsorption capacity was 76 mg/g at 20 °C [36]. In another study by Ozturk et al., brilliant red was removed with Amberlyst A21 and the maximum adsorption capacity, based on Langmuir isotherm, was determined to be 208 mg/g [37]. Rhodamine B was removed by impregnated Dowex 5WX8 cation exchange resin with t-butyl phosphate. By fitting experimental data, 43.47 mg/g maximum adsorption capacity was determined by the Langmuir model [38]. 

##### Freundlich Isotherm

Adsorption experimental data were also investigated using the Freundlich isotherm model. The Freundlich isotherm theory may be applied when the dye adsorption takes place on the active sites of the heterogeneous surface of a resin mass [16]. The logarithmic equation of the Freundlich isotherm is represented by:

(7) $${\mathrm{lnQ}}_{\mathrm{eq}}{=\mathrm{lnK}}_{f}+\frac{1}{n}{\mathrm{lnC}}_{\mathrm{eq}}$$

where: K_f_ and n represent adsorption capacity and intensity for IRA 402(Cl^−^), respectively. Thus, by graphical representation of the lnQe vs. lnCe, the values $\frac{1}{n}\;$ and lnK_f_ from the slope and intercept were calculated by the linear regression equation y = 0.1954 x + 3.2477 as follows: K_f_ = 25.7 mg/g, n = 5.12 and $\;\frac{1}{n}$ = 0.2 (see Table 3). Additionally, $\frac{1}{n}$ is less than 1, representing a measure of adsorption process probability. The R^2^ value of 0.8614 suggests that this model is less adequate in describing the adsorption equilibrium data of AB 113 onto IRA 402(Cl^−^) when compared with the R^2^ obtained using the Langmuir model. A comparison of theoretical models with the experimental data is presented in Figure 4.

### 3.4. Desorption Studies

For ion exchange resin regeneration, it is recommended to use acid solutions [20,39,40]. 

The behavior of regeneration can be explained as follows. (i) The eluent solution does not act on the resin loaded with dye. Thus, the stability of the resin loaded with AB 113 in the presence of the HCl eluent offers a new direction of research through which the newly obtained resins can be used to separate and concentrate metal ions from a wide variety of effluents, especially from acidic ones. A similar study has been presented in the literature in this regard when the anion exchange resin IRA 400 Cl^−^ was impregnated with dithizone functional group. The loaded resin was found to be stable in the presence of a 5 M HCl solution. The authors suggested that the structure of the chelating agent (a sulphonic acid derivative of ditizone) contained two sulfonic groups that favored both ion exchange and physical adsorption [41]. (ii) The effluent solution has the effect of desorption of the colorant retained on the resin. According to the methodology presented in (ii), regenerated resin can be studied in several adsorption and desorption cycles [42].

**Figure 6:**
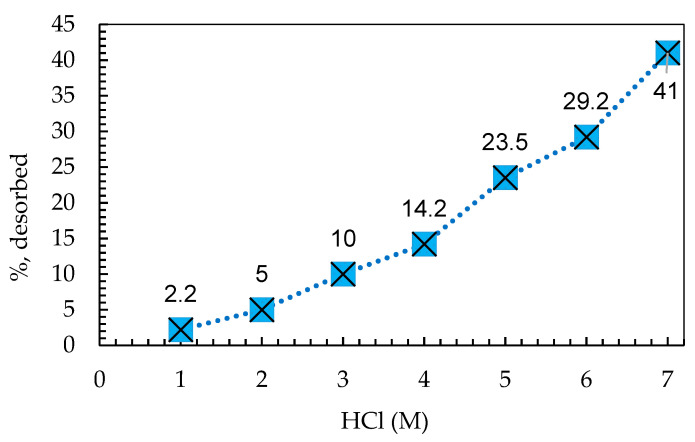
Influence of acidic medium for AB 113 dye desorption of loaded resin. The values presented are mean of three replicates with RSD% below 8% for all experiments.

The resin loaded with AB 113 was subjected to a single adsorption/desorption cycle. In our experiment, when applying the desorption study, the quantity of AB 113 decreased from the IRA 402 resin mass with an increase in HCl concentrations from 1 to 7 M, as presented in Figure 6. Moreover, it is evident that AB 113 continued to be retained quantitatively on the resin mass even when 7 M HCl solutions were used. Considering the acid solutions effect, one may say that ion exchange is not the only mechanism of AB 113 adsorption. The supplementary interactions may be taken into account because only 40% of the AB 113 desorbed from IRA 402 was detected in the 7 M HCl supernatant solution. 

### 3.5. Pilot Laboratory Experiment

**Figure 7:**
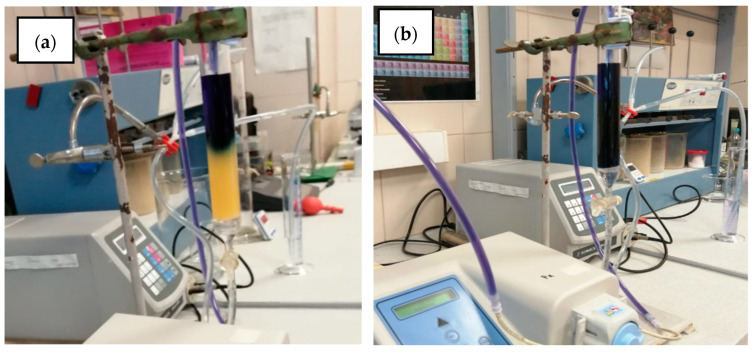
Pilot continuous mode adsorption installation at the National Research and Development Institute for Industrial Ecology (ECOIND) laboratory: (**a**) loaded bed column after 174 h of continuous solution flow; (**b**) loaded bed column after 348 h of continuous solution flow of AB 113.

Successful application of the experimental data was achieved when a pilot laboratory installation was tested. The laboratory pilot installation was developed in order to evaluate the efficiency AB 113 dye removal from inflow solutions onto an IRA 402(Cl^−^) resin bed. The pilot laboratory experiment developed is presented in Figure 7a,b. 

Experimental parameters of the pilot laboratory installation were calculated according to our previous research [43]. The porosity fraction of IRA 402(Cl^−^) bed (φ) was measured as being 0.018 L (18 cm^3^). Based on the above parameters, the hydraulic retention time (HRT) was established as:

(8) $$\mathrm{HRT}=\frac{\varphi \cdot \mathrm{Hb}}{Q}$$

HRT = 25.7 min. The horizontal surface area

(9) $$S=\frac{\pi }{4}{Ø}^{2}$$

S = 4.91 cm^3^ was also calculated. In addition, the total volume of the column:

Vt = S × H(10)

Vt = 4.91 × 25; Vt = 123 cm^3^ and working volume:

Vw = S × Hb
(11)

Vw = 4.91 × 10; Vw = 49.1 cm^3^ were also calculated. The flow rate of AB 113 solutions through the IRA 402(Cl^−^) resin bed was calculated as

(12) $$V=\frac{Q}{S}$$

**Figure 8:**
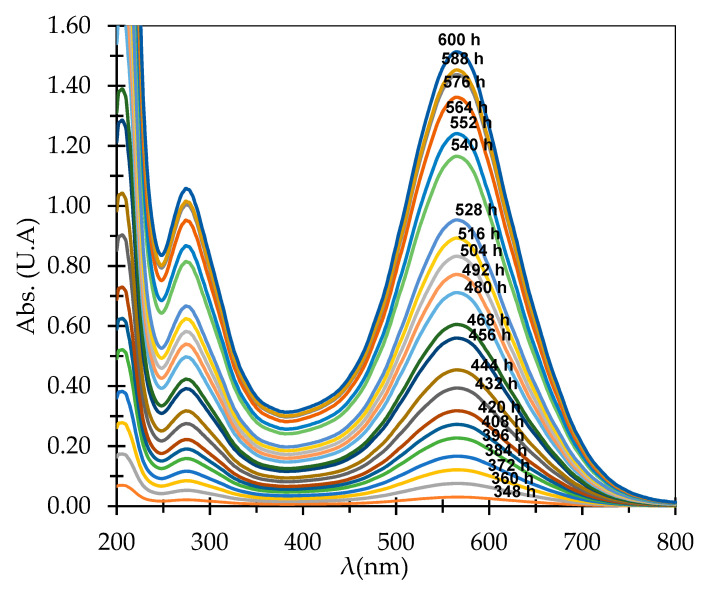
UV-Vis spectra of AB 113 dye existing in outflow samples.

**Figure 9:**
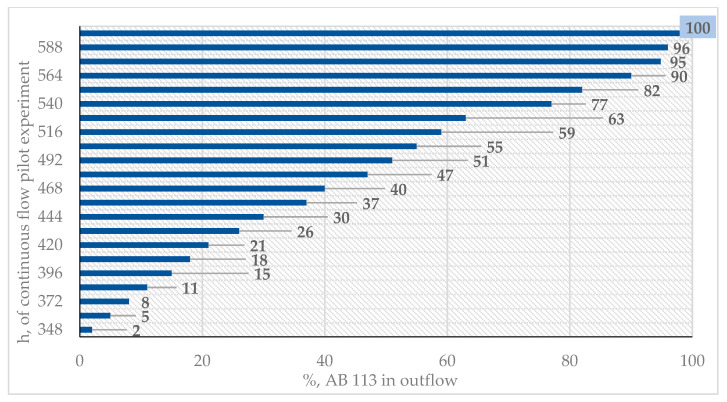
Graphical representation of AB 113 dye existing in outflow.

V = 0.074($\frac{m}{h}$). Samples were taken after 12 h and the inflow and outflow concentrations of AB 113 were determined using spectrometric equipment, see Figure 8. From the outflow, samples were collected for 600 h (25 days) and 50 effluent samples were taken every 12 h. The AB 113 dye was completely removed by the resin bed after 336 h. After, 348 h, the percentage of AB 113 in effluent was detected over the determination limit of the analytical method, with the behavior presented in Figure 8 and Figure 9. After 600 h, saturation of the resin bed was observed, with the initial concentration of inflow being equal to the outflow concentration. Awasthi and Datta presented a column through which 10,000 L/day of textile effluent had been treated by applying a column experiment. Characteristics of the column were: 0.1127 m ID, 0.0199 m height containing 0.066 kg of Amberlite XAD-7HP resin impregnated with Aliquat 336 [44]. Sinha et al. studied the removal of Congo Red Dye using a fixed bed of Amberlite IRA 400 with continuous flow. The service time and bed depth service parameters were studied in order to optimized the process [23].

### 3.6. Enzymatic Bio-Degradation of the AB 113 Dye 

**Figure 10:**
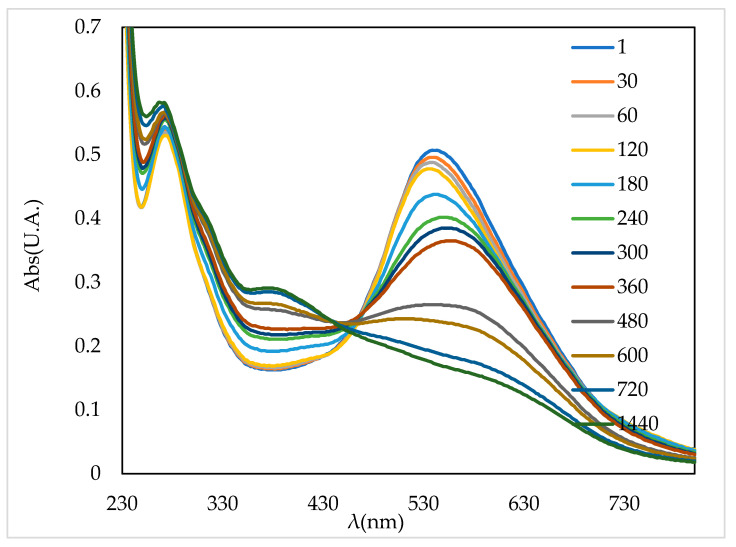
UV-Vis spectra of AB 113 biodegradation in presence of laccase.

**Figure 11:**
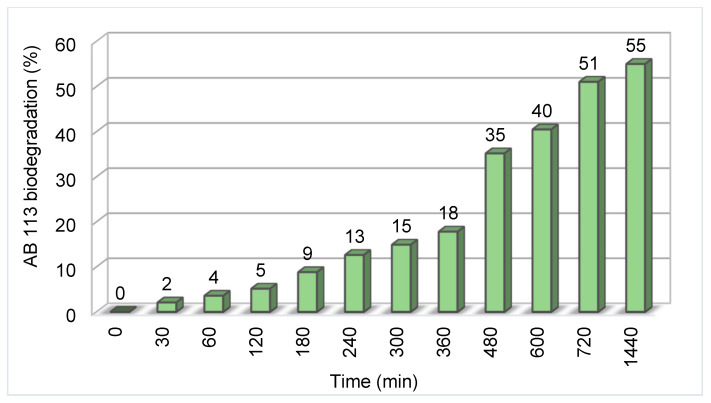
Time influence on AB 113 Laccase biodegradation effect.

Modern depollution technology should follow sustainable development requirements. Thus, biotechnologies based on using microbes or enzymes are an important evolving field. In our previous study [16] it was observed that the best gryphalan navy blue A193 azo dye enzymatic degradation was obtained at pH = 4. Considering this result, the laccase biodegradation of AB 113 was evaluated only at pH = 4. The biodegradation efficiency was calculated by monitoring the surface area of UV-VIS spectrum in the 450–750 nm range between 1 to 1440 min compared to the control containing dye AB 113 without laccase (Figure 10). During the experimental study the biodegradation rate increased up to 35% in 8 h of incubation, reaching a maximum degradation at 24 h when more than 55% of the 0.00003 M AB 113 concentration was bio-degraded (Figure 11).

### 3.7. TG Analysis of AB 113 Dye

**Figure 12:**
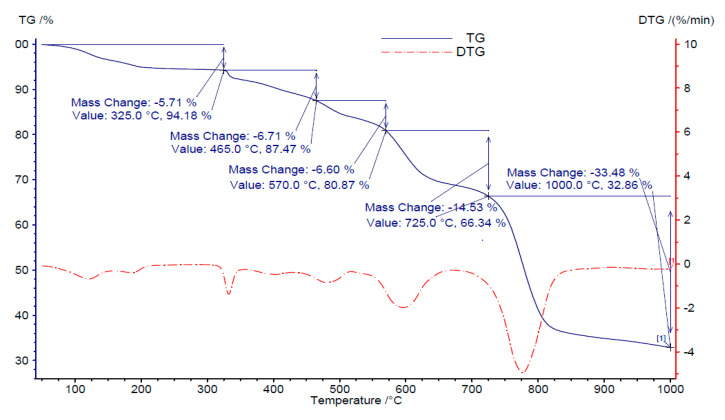
TG and DTG curves of AB 113 dye.

Thermal analysis techniques are frequently used in order to obtain useful information concerning the composition and stability of different species such organic azo dye [45]. In order to obtain such information, the thermal behavior of AB 113 was investigated by TG analysis. The TG and DTG curves corresponding to AB 113 dye thermal behavior indicate that its decomposition in argon atmosphere follows several steps. The first step constitutes the release of water molecules up to 150 °C. The anhydrous species then undergo gradual degradation. According to the data the residual mass formed at 800 °C is 32.86%, as presented in Figure 12.

### 3.8. FTIR Spectroscopy Results

**Figure 13:**
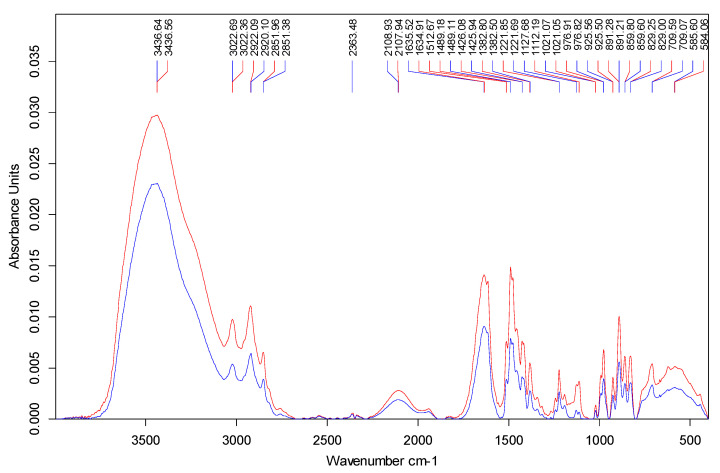
FTIR spectra of pure IRA 402(Cl^−^) resin (red line) and of AB113 loaded resin (blue line).

Spectra of pure IRA 402(Cl^−^) resin and of AB113-loaded resin are shown in Figure 13.

One may observe only very small modifications of peak positions in the 1 to 2 cm^−1^ range, for the C–H stretching vibration at 2922 cm^−1^, C=C stretching at 1615 cm^−1^ and C–N stretching at 1126 cm^−1^ [46]. The above changes in the peak positions are correlated with the ionic interactions taking place between the quaternary ammonium methyl groups and the dye, also with π–π and other van der Waals interactions. The broad intense peak of 3436 cm^−1^ attributed to hydroxyl group and the peak at 1426 cm^−1^ attributed to the CH2 bending vibrations of the pure resin conserve their position after contaminant deposition showing that the hydrocarbon skeleton does not take part to the adsorption process and the hydroxyl group vibration frequency is not modified by the adsorbent–adsorbate interactions as found in the literature [47]. The specific BET surface area and average pore diameter of Amberlite 402 IRA is 2.45 m^2^/g and 1.89 nm allowing retention of high-molecular-weight compounds as reported [47]. After amination, the specific surface areas of the resins and the average pore diameter decreases [48].

## 4. Conclusions

All compounds containing ionizable groups in their structure may be retained in solution by an ion exchange mechanism, regardless of their concentration. Azo dyes’ ionic groups ensure their solubility and the possibility of the ion exchange mechanism retention on materials with ion exchange properties. Thus, AB 113 azo dye could be separated from solution using polymeric anion exchange resin IRA 402 in the batch study. In addition, the time required to reach the adsorption equilibrium was 90 min. Due to the shape and the bulky structure of the AB 113 dye, it could be more difficult to reach the functional groups on the resin structure. The results showed that the adsorption kinetics of AB 113 dye in anion-exchange resin is best described by PFO, compared to PSO and the Weber–Morris model. The ion exchange equilibrium was characterized by operational parameters that demonstrated that the AB 113 retention on the IRA 402 is efficient. The adsorption isotherms study indicated that the ion exchange equilibrium between the IRA 402 resin and AB 113 is best described by the Langmuir model. Taking into account the hypothesis of the Langmuir model, evaluating the data obtained at equilibrium, it was found that the adsorption process takes places by active ion exchange centers at the surface of the resin. The maximum adsorption capacity of the adsorbent monolayer was found to be 130 mg/g, applying the Langmuir isotherm model. All experimental parameters confirm a strong interaction between AB 113 and the IRA 402(Cl^−^), controlled mainly by the ion exchange equilibrium. The percentages of AB 113 eluted from the loaded IRA 402 at 1 M and 3 M HCl were enhanced only up to 10%. High volumes of aqueous solutions treated in continuous flux mode suggest that the IRA 402 may be used in dye-polluted wastewater treatment. Degradation of AB 113 dye under the influence of laccase at pH = 4 was monitored in the last part of this study. Moreover, it was proved that the enzymatic degradation enriches the wastewater treatment process, when the column bed is exhausted.

## Figures and Tables

**Table 1:** Kinetic constants describing the rate of adsorption of AB 113 dye onto the IRA 402(Cl^−^).

Lagergren Model (PFO)	K_1_ (min^−1^)	Q_t_ (mg/g)	R^2^
	0.02	162.7	0.9275
Morris–Weber Model First stage Second stage	K_id_ (min^−1^)	C	
	24	114	0.9918
	2.59	76.1	0.6504
Pseudo Second Order (PSO) Model	K_2_ g/(mg min)	Q_t_ (mg/g)	
Type 1	0.0300	626	0.0300
Type 2	0.0001		0.8577
Type 3	0.0007	27.9	0.1563
Type 4	0.0446	0.07	0.1563

Note: The experimental adsorption capacity at 135 min was Q_t_ = 104.5 mg/g.

**Table 3:** Langmuir and Freundlich parameters for AB 113 adsorption onto IRA 402 mass.

	Langmuir Isotherm Model			Freundlich Isotherm Model			
T (°C)	Q_m_ (mg/g)	b (L/mg)	R^2^	K_f_ (mg/g)	n	$\frac{1}{n}$	R^2^
25 ± 2	130	0.01	0.9575	25.7	5.12	0.20	0.8614

## Data Availability

The data supporting reported results are available on request from the authors.

## Supplementary Materials

The following are available online at https://www.mdpi.com/article/10.3390/polym13223991/s1, Figure S1: Fitting kinetics results for PSO models modulated as: t/Q_t_ vs. t (a); 1/Q_t_ vs. 1/t (b); Q_t_ vs. Q_t_/t (c) and Q_t_/t vs. Q_t_ (d).

## References

[1] Katheresan V., Kansedo J., Lau S.Y. (2018). Efficiency of various recent wastewater dye removal methods: A review. J. Environ. Chem. Eng..

[2] De Gisi S.G., Lofrano M., Grassi M.N. (2016). Characteristics and adsorption capacities of low-cost sorbents for wastewater treatment: A review. Sustain. Mater. Technol..

[3] Mojsov K.D., Andronikov D., Janevski A., Kuzelov A., Gaber S. (2016). The application of enzymes for the removal of dyes from textile effluents. Adv. Technol..

[4] Nguyen T.A., Juang R.-S. (2013). Treatment of waters and wastewaters containing sulfur dyes: A review. Chem. Eng. J..

[5] Robinson T., McMullan G., Marchant R., Nigam P. (2001). Remediation of dyes in textile effluent: A critical review on current treatment technologies with a proposed alternative. Bioresour. Technol..

[6] Yagub M.T., Sen T.K., Afroze S., Ang H.M. (2014). Dye and its removal from aqueous solution by adsorption: A review. Adv. Colloid Interface Sci..

[7] Zeng G., Ye Z., He Y., Yang X., Ma J., Shi H., Feng Z. (2017). Application of dopamine-modified halloysite nanotubes/PVDF blend membranes for direct dyes removal from wastewater. Chem. Eng. J..

[8] Kant R. (2012). Textile dyeing industry an environmental hazard. Sci. Res..

[9] Forgacs E., Cserhati T., Oros G. (2004). Removal of synthetic dyes from wastewaters: A review. Environ. Int..

[10] Joshi M., Bansal R., Purwar R. (2004). Colour removal from textile effluents. Indian J. Fibre Text. Res..

[11] Peng Y., Zhang Y., Huang H., Zhong C. (2018). Flexibility induced high-performance MOF-based adsorbent for nitroimidazole antibiotics capture. Chem. Eng. J..

[12] Montoya-Suarez S., Colpas-Castillo F., Meza-Fuentes E., Rodríguez-Ruiz J., Fernandez-Maestre R. (2016). Activated carbons from waste of oil-palm kernel shells, sawdust and tannery leather scraps and application to chromium (VI), phenol, and methylene blue dye adsorption. Water Sci. Technol..

[13] Liu L., Zhang J., Tan Y., Jiang Y., Hu M., Li S., Zhai Q. (2014). Rapid decolorization of anthraquinone and triphenylmethane dye using chloroperoxidase: Catalytic mechanism, analysis of products and degradation route. Chem. Eng. J..

[14] Wang Z., Xue M., Huang K., Liu Z. (2011). Textile dyeing wastewater treatment. Adv. Treat. Text. Effl..

[15] Marin N.M., Pascu L.F., Demba A., Nita-Lazar M., Badea I.A., Aboul-Enein H. (2019). Removal of the Acid Orange 10 by ion exchange and microbiological methods. Int. J. Environ. Sci. Technol..

[16] Marin N.M., Tiron O., Pascu L.F., Costache M., Nita-Lazar M., Badea I.A. (2018). Synergistic methodology based on ion exchange and biodegradation mechanisms applied for metal complex dye removal from waste waters. Rev. Chim..

[17] Helfferich F.G. (1995). Ion Exchange.

[18] Golden I.E., Wilson I.D. (2000). Encyclopedia of Separation Science.

[19] Acikara Ö.B. (2013). Ion exchange chromatography and its applications. Column Chromatogr..

[20] Cyganowski P., Dzimitrowicz A. (2020). A Mini-Review on Anion Exchange and Chelating Polymers for Applications in Hydrometallurgy, Environmental Protection, and Biomedicine. Polymers.

[21] Polska-Adach E., Wawrzkiewicz M., Hubicki Z. (2019). Removal of acid, direct and reactive dyes on thepolyacrylic anion exchanger. Physicochem. Probl. Miner. Process..

[22] Rashed M.N. (2013). Adsorption technique for the removal of organic pollutants from water and wastewater. Org. Pollut.-Monit. Risk Treat..

[23] Sinha S., Behera S., Das S., Basu A., Mohapatra R., Murmu B., Dhal N., Tripathy S., Parhi P. (2018). Removal of Congo Red dye from aqueous solution using Amberlite IRA-400 in batch and fixed bed reactors. Chem. Eng. Commun..

[24] Bulgariu D., Nacu G., Măluan T., Bulgariu L. (2016). Kinetic study of lead (II) removal from aqueous solution onto lignin-based materials. Cellul. Chem. Technol..

[25] Shirzad-Siboni M., Jafari S.J., Giahi O., Kim I., Lee S.-M., Yang J.-K. (2014). Removal of acid blue 113 and reactive black 5 dye from aqueous solutions by activated red mud. J. Ind. Eng. Chem..

[26] Jain S.N., Gogate P.R. (2017). Acid Blue 113 removal from aqueous solution using novel biosorbent based on NaOH treated and surfactant modified fallen leaves of Prunus Dulcis. J. Environ. Chem. Eng..

[27] Lee L.Y., Gan S., Tan M.S.Y., Lim S.S., Lee X.J., Lam Y.F. (2016). Effective removal of Acid Blue 113 dye using overripe Cucumis sativus peel as an eco-friendly biosorbent from agricultural residue. J. Clean. Prod..

[28] Gupta V.K., Gupta B., Rastogi A., Agarwal S., Nayak A. (2011). A comparative investigation on adsorption performances of mesoporous activated carbon prepared from waste rubber tire and activated carbon for a hazardous azo dye—Acid Blue 113. J. Hazard. Mater..

[29] Greluk M., Hubicki Z. (2011). Efficient removal of Acid Orange 7 dye from water using the strongly basic anion exchange resin Amberlite IRA-958. Desalination.

[30] Wawrzkiewicz M., Hubicki Z. (2009). Removal of tartrazine from aqueous solutions by strongly basic polystyrene anion exchange resins. J. Hazard. Mater..

[31] Zhu Z., Zhang M., Liu F., Shuang C., Zhu C., Zhang Y., Li A. (2016). Effect of polymeric matrix on the adsorption of reactive dye by anion-exchange resins. J. Taiwan Inst. Chem. Eng..

[32] Greluk M., Hubicki Z. (2011). Comparison of the gel anion exchangers for removal of Acid Orange 7 from aqueous solution. Chem. Eng. J..

[33] Wawrzkiewicz M., Hubicki Z. (2010). Equilibrium and kinetic studies on the sorption of acidic dye by macroporous anion exchanger. Chem. Eng. J..

[34] Greluk M., Hubicki Z. (2013). Evaluation of polystyrene anion exchange resin for removal of reactive dyes from aqueous solutions. Chem. Eng. Res. Des..

[35] Yang K., Xing J., Xu P., Chang J., Zhang Q., Usman K.M. (2020). Activated Carbon Microsphere from Sodium Lignosulfonate for Cr(VI) Adsorption Evaluation in Wastewater Treatment. Polymers.

[36] Kumar S.V. (2019). Adsorption kinetics and isotherms for the removal of rhodamine B dye and Pb + 2 ions from aqueous solutions by a hybrid ion-exchanger. Arab. J. Chem..

[37] Ozturk G., Silah H. (2020). Adsorptive Removal of Remazol Brilliant Blue R from water by using a macroporous polystyrene resin: Isotherm and kinetic studies. Environ. Process..

[38] Khan M.A., Siddiqui M.R., Otero M., Alshareef S.A., Rafatullah M. (2020). Removal of Rhodamine B from Water Using a Solvent Impregnated Polymeric Dowex 5WX8 Resin: Statistical Optimization and Batch Adsorption Studies. Polymers.

[39] Patel H. (2021). Review on solvent desorption study from exhausted adsorbent. J. Saudi Chem. Soc..

[40] Terangpi P., Chakraborty S. (2017). Adsorption kinetics and equilibrium studies for removal of acid azo dyes by aniline formaldehyde condensate. Appl. Water Sci..

[41] Tanaka H., Chikuma M., Harada A., Ueda T., Yube S. (1976). A new chelate-forming resin with dithizone functional group prepared by the conversion of an anion-exchange resin. Talanta.

[42] Jia Y., Ding L., Ren P., Zhong M., Ma J., Fan X. (2020). Performances and Mechanism of Methyl Orange and Congo Red Adsorbed on the Magnetic Ion-Exchange Resin. J. Chem. Eng. Data.

[43] Marin N.M., Dinu L., Stanculescu I., Cristea N.I., Ionescu A.I. (2021). Maize Stalk Material for On-Site Treatment of Highly Polluted Leachate and Mine Wastewater. Materials.

[44] Awasthi A., Datta D. (2019). Application of Amberlite XAD-7HP resin impregnated with Aliquat 336 for the removal of Reactive Blue-13 dye: Batch and fixed-bed column studies. J. Environ. Chem. Eng..

[45] Leulescu M., Rotaru A., Pălărie I., Moanţă A., Cioateră N., Popescu M., Morîntale E., Bubulică M.V., Florian G., Hărăbor A. (2018). Tartrazine: Physical, thermal and biophysical properties of the most widely employed synthetic yellow food-colouring azo dye. J. Therm. Anal. Calorim..

[46] Stanculescu I., Mandravel C., Landy D., Woisel P., Surpateanu G. (2003). Complexation of tetrandrine with calcium ion probed by various spectroscopic methods and molecular modeling. J. Mol. Struct..

[47] Jachuła J., Hubicki Z. (2013). Removal of Cr(VI) and As(V) ions from aqueous solutions by polyacrylate and polystyrene anion exchange resins. Appl. Water Sci..

[48] Liu W., Zhang Y., Wang S., Bai L., Deng Y., Tao J. (2021). Effect of Pore Size Distribution and Amination on Adsorption Capacities of Polymeric Adsorbents. Molecules.

