# Supplementary material for: Application of Amberlite IRA 402 Resin Adsorption and Laccase Treatment for Acid Blue 113 Removal from Aqueous Media
Source: Polymers (Basel). 2021 Nov 18;13(22):3991. doi: 10.3390/polym13223991 (PMC8621439; doi:10.3390/polym13223991)
Supplement: Supplementary file 1 [file polymers-13-03991-s001.zip › polymers-1446015-supplementary.pdf]

Supplementary Material

Application of Amberlite IRA 402 resin Adsorption and Laccase Treatment for Acid Blue 113 Removal from Aqueous Media

Nicoleta Mirela Marin[1*] and Ioana Stanculescu[2-4*]

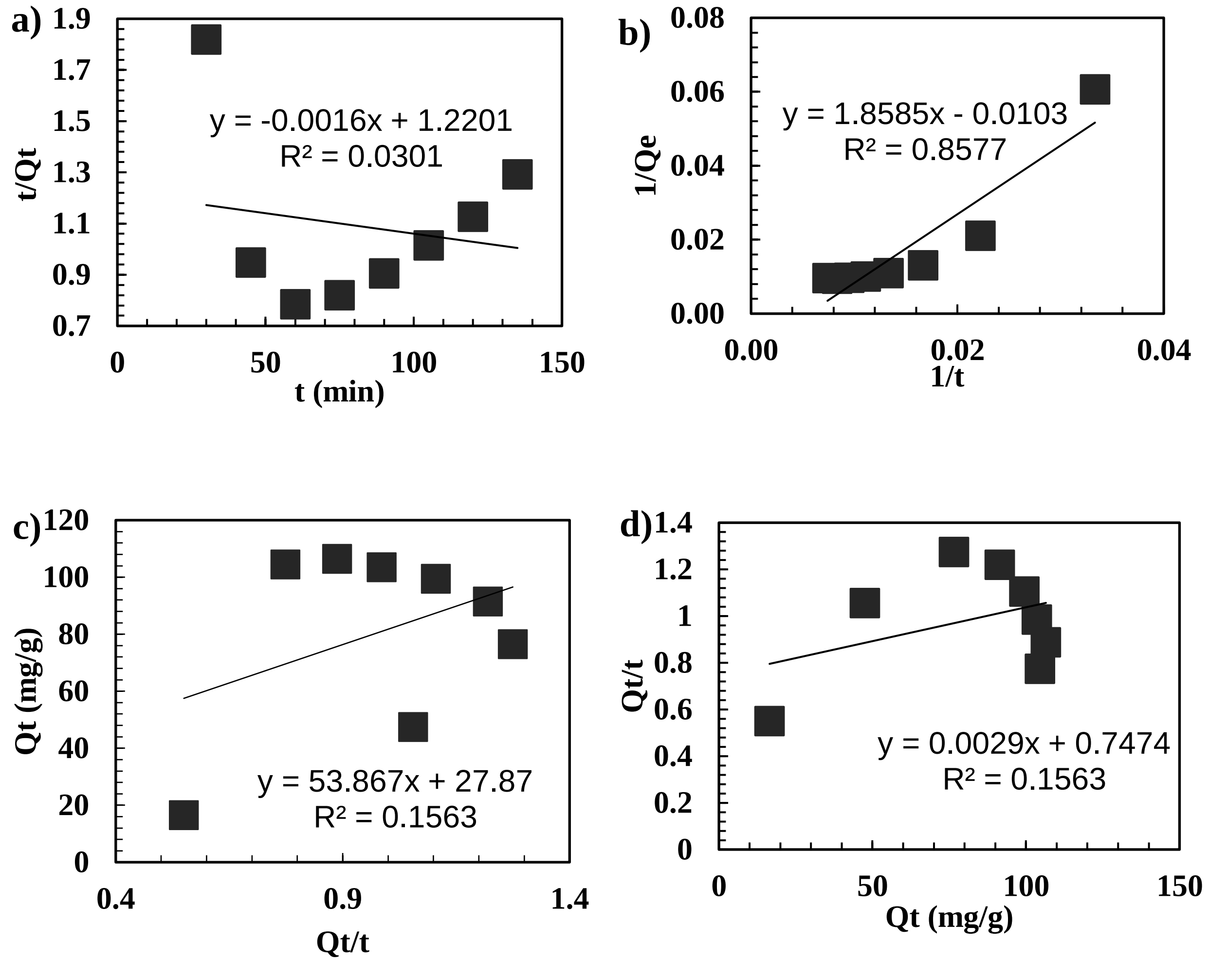

Figure S1. Fitting kinetics results for PSO models modulated as: t/Q_t vs t (a); $1/Q_t$ vs 1/t (b); Q_t vs Q_t/t (c) and Q_t/t vs Q_t (d)
